# A comparison of bone density and bone morphology between patients presenting with hip fractures, spinal fractures or a combination of the two

**DOI:** 10.1186/1471-2474-14-68

**Published:** 2013-02-22

**Authors:** Richard G Crilly, Lizebeth Cox

**Affiliations:** 1 Division of Geriatric Medicine, Faculty of Medicine, University of Western Ontario, London, ON, Canada; 2 Faculty of Health Sciences, University of Western Ontario, Windermere Road, London, ON, Canada; 3 St Joseph’s Health Care London, 801 Commissioners Road East, London, ON, N6C 5J1, Canada

**Keywords:** Hip fracture, BMD, Trabecular and cortical bone, Vertebral compression fractures

## Abstract

**Background:**

Currently it is uncertain how to define osteoporosis and who to treat after a hip fracture. There is little to support the universal treatment of all such patients but how to select those most in need of treatment is not clear. In this study we have compared cortical and trabecular bone status between patients with spinal fractures and those with hip fracture with or without spinal fracture with the aim to begin to identify, by a simple clinical method (spine x-ray), a group of hip fracture patients likely to be more responsive to treatment with current antiresorptive agents.

**Methods:**

Comparison of convenience samples of three groups of 50 patients, one with spinal fractures, one with a hip fracture, and one with both. Measurements consist of bone mineral density at the lumbar spine, at the four standard hip sites, number, distribution and severity of spinal fractures by the method of Genant, cortical bone thickness at the infero-medial femoral neck site, femoral neck and axis length and femoral neck width.

**Results:**

Patients with spinal fractures alone have the most deficient bones at both trabecular and cortical sites: those with hip fracture and no spinal fractures the best at trabecular bone and most cortical bone sites: and those with both hip and spinal fractures intermediate in most measurements. Hip axis length and neck width did not differ between groups.

**Conclusion:**

The presence of the spinal fracture indicates poor trabecular bone status in hip fracture patients. Hip fracture patients without spinal fractures have a bone mass similar to the reference range for their age and gender. Poor trabecular bone in hip fracture patients may point to a category of patient more likely to benefit from therapy and may be indicated by the presence of spinal fractures.

## Background

In the evaluation of medications for osteoporosis the essential requirement is the prevention of fractures. Although initially the focus was on the prevention of compression fractures of the spine, the importance of preventing non-spinal fractures has emerged and is now a critical expectation of the drugs. Without exception, however, the therapies are better at preventing spinal fractures than non-spinal fractures, possibly because they are more effective on trabecular bone than cortical bone, the latter being an important contributor to the strength of the non-spinal fracture sites [1]. Despite this, the focus has shifted from osteoporosis as a disease of trabecular bone resulting in spinal fractures, to a state of risk of fracture, with a focus on non-vertebral, substantially cortical bone fractures that occur in a fall. The non-vertebral fractures now dominate the fracture risk assessment paradigms which are based on measures which better reflect cortical bone mass, specifically femoral neck density [2,3].

By and large, it seems that patients at the lower end of the normal distribution of bone densities in the general population, are, should they fall, at greater risk of fracture than those at the upper end of the distribution. For example, in the EPIDOS study of women average age of 80 years, those with BMD on heel ultrasound and neck of femur BMD that were below average had a risk of hip fracture of 1.96% per year compared to 0.27% if above average [4].

In addition to simple bone thinning with age, some architectural features of the neck of femur might be important in determining risk of hip fracture. Differences in hip bone density, mostly at the neck of femur site, and differences in architecture (Singh index) and geometry (hip axis length, neck-shaft angle) of the proximal femur between those with a hip fracture and normal controls have been described [5-9]. An additional issue is the design of the bone and whether it is being exposed to a force for which it was not designed [10]. Thus the trabecular pattern of the femur is arranged to withstand loading from above and not to withstand a blow on the greater trochanter from the side, as occurs in falls in the elderly and which generates a force that greatly exceeds the strength of any hip [11]. It is likely that many of the structural differences between hip fracture patients and others will be unresponsive to treatment with current osteoporosis medications.

By contrast, spinal fractures do not usually require a fall and can be sustained in normal activity. Indeed, the vertebral fracture is to some extent distinguished from the non-vertebral fracture in that it usually occurs under a force the vertebra should have been able to withstand, in contrast to the non-vertebral fracture where more is expected of the bone than it was designed to meet. Nonetheless, these non-vertebral fractures have come to be considered fragility fractures, and have come to be considered indicative of osteoporosis, although they are best predicted by a measure of cortical bone. Nonetheless, most of the clinical trials, predominantly with bisphosphonates, were done on people many or most of whom had spinal compression fractures and better fitted the trabecular bone loss model. These patients displayed anything up to 13% chance of a new spinal fracture within a year, higher if there had been a recent compression fracture, but a lesser degree of increased risk of non-vertebral fracture [12]. Thus it appears that the loss of trabecular bone puts the spine particularly at risk, but only partly compromises the non-vertebral sites. Consistently, the fractures which respond best to treatment are the spinal fractures of trabecular bone. Possibly the degree of trabecular bone deficiency at a specific site may determine the degree of response that can be expected from treatment.

The question arises, when does the spinal fracture syndrome merge into or overlap with the mechanical model of low trauma fracture? Is there, at one end of the scale, the patient with a hip fracture who has deficient trabecular bone while at the other end the patient with perhaps some loss of cortical bone but in whom the fracture is mostly traumatic and where response to treatment is likely to be minimal? In other words, does the degree of trabecular bone deficiency indicate a state more likely to respond to treatment and how can these patients be identified? If, for example, hip fracture patients have compression fractures, is this on the basis of low trabecular bone mass, or are these also largely traumatic? If the former, this may point to a state of deficient trabecular bone mass throughout the skeleton, but if largely traumatic or mechanical in nature, bone mass may not be much reduced but we might expect a different distribution of spinal fractures, more being low in the lumbar spine, the site most vulnerable in a fall or accident [13].

To begin to explore this possibility we have, in this study, compared, on the basis of trabecular and cortical measurements, three approximately age-matched groups of older patients with osteoporotic-type fractures consisting of; patients attending an out-patient osteoporosis clinic referred because of the presence of compression fractures (but with no history of hip fracture); patients admitted to a hip fracture rehabilitation program who, upon spinal x-ray, were found to have compression fractures; and similar patients found not to have compression fractures. The first group is proposed as the gold standard for the presence of severe trabecular bone loss, the second, hip and spinal fracture patients as potentially having trabecular osteoporosis (as the spinal fracture could, itself, be traumatic and caused by falling) and the third, those with only a hip fracture, as potentially non-osteoporotic traumatic fracture patients.

## Methods

### Patient selection

This was a retrospective data review of patients mostly over the age of 70 years presenting to an osteoporosis clinic with spinal compression fractures but no history of hip fracture and those admitted to a rehabilitation unit following a hip fracture. All patients had a standard osteoporosis series of radiographs performed, consisting of a lateral and AP view of the thoracic and lumbar spine and AP view of the pelvis, all in digital form. All patients underwent bone mineral density at spine and hip sites. Hip fracture patients from long term care, and those who are admitted directly to LTC from the orthopedic unit, are not transferred to the rehabilitation program and are not represented in this study. These patients are older than the population studied here. Likewise, younger patients who can be discharged directly home following surgery do not come to the rehabilitation program and are also not represented here.

For this study there are a total of 150 patients comprising 50 patients from each of three patient groups, viz. patients from the osteoporosis clinic with compression fractures and no history of hip fracture, patients from the hip fracture rehabilitation population who were found to have spinal compression fractures on x-ray, and patients with a hip fracture but no spinal fracture. Patients were required to have had: (1) BMD; (2) radiographic images of the thoracic and lumbar spine; and (3) radiographic image of the pelvis with at least one clean hip joint (no prosthetic or replacement device).

Several variables were examined as follows:

#### BMD

The BMD for the spine (L2-L4), the total hip, the intertrochanteric region, the trochanteric and the neck of the proximal femur were recorded. All BMDs were obtained using dual-energy x-ray absorptiometry (DEXA). For each region tested the BMD value (g/cm2), t-score and z-score were recorded. The z-score is the degree in standard deviation units by which the individual differs from the mean level for their age and sex as compared to the normative data base for the specific BMD machine. We use this comparison to determine if the individuals and groups are similar or otherwise to the normal population. The spine site is predominantly a measure of trabecular bone (66%), the neck of femur site predominantly cortical bone (75%) while the intertrochanteric site is about 50% trabecular and 50% cortical [1]. In the following we refer to the spine as a trabecular bone site and the neck of femur as a cortical bone site though none is exclusively one or the other.

#### The location and severity of vertebral fractures

Radiographic images of the lateral vertebral column were recorded, graded, and analyzed based on Genant et al.’s semiquantitative assessment of vertebral fractures [14]. This method assesses the presence and severity of a fracture by visual determination. The degree of height reduction determines the grade (0, 1, 2 or 3) assigned to each vertebra from T4 to L4.

The Spinal Fracture Index used Genant’s semiquantitative assessment for each vertebrae from T4-L4 [14]. The radiographic image of each vertebrae is visually assessed and given a grade based on the level of deformity (Grade 0 = No visible deformity; Grade 1 = 20-25% deformity; Grade 2 = 25-40% deformity; Grade 3 = more than 40% deformity). The scores for each vertebrae from T4-L4 are then summed and divided by 13 to get an average SFI for the patient. The Spinal Deformity Index (SDI) is derived from Genant’s semiquantitative assessment to describe the total fracture status of the spine. The SDI is calculated for each patient by summing the vertebral fracture grades (0, 1, 2 or 3) from T4 to L4 (14).

As spinal BMD is influenced by the presence of spinal degenerative disease, we recorded this when it was provided by the formal radiology report which was done by radiologists unaware of this study.

Hip axis length, femoral neck length and femoral neck cortical bone thickness were measured as outlined by Peacock and colleagues [5]. Briefly, the femoral axis length measures from the lateral point of the femur where the greater trochanter joins the shaft to the centre apex of the femoral head. The hip axis length extends that measurement to the inner (pelvic) surface of the acetabulum. Femoral neck width was measured in accordance with that reported by Karlsson and colleagues and is the narrowest point of the femoral neck [15].

Statistical analysis was performed using IBMSPSS Statistics Software version 19. The SPSS Test of Normality employed was the Shapiro-Wilk Test which indicated normality for all parameters except trochanteric BMD, femoral neck BMD, femoral neck width and hip axis length for which the Mann–Whitney *U* test was used to compare groups rather than the *t*-test used for the others.

Ethics approval was obtained through the University of Western Ontario Ethics Board.

## Results

Table 1 outlines the characteristics of patients presenting to a specialized osteoporosis clinic with compression fractures (N=50; mean age = 76.56+/−9.34 years; 14 men) and those of similar age and gender presenting with hip fracture (both intertrochanteric and subcapital types) half of whom were selected for the presence of compression fractures (N=50; mean age =79.46+/−6.45 years; 12 men) and half for their absence (N=50; mean age=81.00+/−5.89 years; 9 men).

**Table 1:** Comparison of the three groups

**Characteristic**	**Only spinal fractures**	**Spinal & hip fractures**	**Only hip fractures**	**All hip fractures**
	**(N=50)**	**(N=50)**	**(N=50)**	**(N=100)**
Women	36	38	41	79
Men	14	12	9	21
Age, mean +/- SD	76.6+/- 9.3^+#^	79.5+/- 6.5	81.0+/- 5.9^+^	80.2+/- 6.2^#^
Age, Range	52-92	65-91	66-89	65-91
Lumbar BMD Results, mean +/- SD	0.801+/-0.178^*+#^	0.913+/-0.176^*^	0.967+/-0.205^+^	0.940+/-0.192^#^
Lumbar T-Score, mean +/- SD	-2.539+/-1.551^*+#^	-1.578+/-1.542^*^	-0.956+/-1.832^+^	-1.265+/-1.713^#^
Lumbar Z-Score, mean +/- SD	-0.461+/-2.003^*+#^	0.533+/-1.442^*^	1.233+/-2.029^+^	0.864+/-1.767^#^
Total Hip BMD Results, mean +/- SD	0.666+/-0.174	0.703+/-0.143	0.727+/-0.136	0.716+/-0.139
Total Hip T-Score, mean +/- SD	-2.251+/-1.457	-1.946+/-1.319	-1.866+/-0.999	-1.905+/-1.163
Total Hip Z-Score, mean +/- SD	-0.577+/-1.286	-0.420+/-1.128	-0.102+/-1.012	-0.268+/-1.078
Intertrochanteric Hip BMD Results, mean +/- SD	0.773+/-0.211^+#^	0.825+/-0.164	0.864+/-0.167^+^	0.845+/-0.166^#^
Intertrochanteric Hip T-Score, mean +/- SD	-2.168+/-1.261^+#^	-1.855+/-0.979	-1.552+/-0.890^+^	-1.703+/-0.942^#^
Intertrochanteric Hip Z-Score, mean +/- SD	-0.646+/-1.309^+^	-0.421+/-1.076^~^	0.142+/-0.999^+~^	-0.152+/-1.070
Trochanteric BMD Results, mean +/- SD	0.510+/-0.145	0.547+/-0.142	0.556+/-0.133	0.552+/-0.137
Trochanteric T-Score, mean +/- SD	-2.026+/-1.278	-1.686+/-1.188	-1.573+/-1.192	-1.630+/-1.184
Trochanteric Z-Score, mean +/- SD	-0.614+/-1.322^+^	-0.353+/-1.098	0.035+/-1.106^+^	-0.168+/-1.111
Femoral Neck BMD Results, mean +/- SD	0.561+/-0.125	0.590+/-0.103	0.607+/-0.122	0.599+/-0.112^#^
Femoral Neck T-Score, mean +/- SD	-2.681+/-1.057^*#^	-2.177+/-1.245^*^	-2.273+/-0.935	-2.225+/-1.095^#^
Femoral Neck Z-Score, mean +/- SD	-0.622+/-0.910^+^	-0.450+/-1.027	-0.077+/-1.044^+^	-0.272+/-1.044
Hip Axis Length, mm, mean +/- SD	126.91+/-12.55	129.52+/-11.03	128.84+/-11.48	129.18+/-11.21
Femoral Neck Length, mm, mean +/- SD	111.06+/-9.97	112.66+/-8.67	112.43+/-9.00	112.55+/-8.79
Femoral Neck Width, mm, mean +/- SD	37.93+/-4.49	38.77+/-4.01	38.15+/-3.86	38.46+/-3.93
Cortical Bone Thickness, mm, mean +/- SD	4.09+/-0.75^*+#^	4.77+/-0.95^*^	4.55+/-1.15^+^	4.66+/-1.06^#^

* indicates significant differences (p < 0.05) between the 'Only Spinal Fractures' group and the 'Spinal and Hip Fractures' group.

+ indicates significant differences (p < 0.05) between the 'Only Spinal Fractures' group and the ‘Only Hip Fractures' group.

# indicates significant differences (p < 0.05) between the 'Only Spinal Fractures' group and the ‘All Hip Fracture’ group.

~ indicates significant differences (p < 0.05) between the ‘Spinal and Hip Fractures' group and the ‘Only Hip Fractures' group.

### BMD

**Figure 1:**
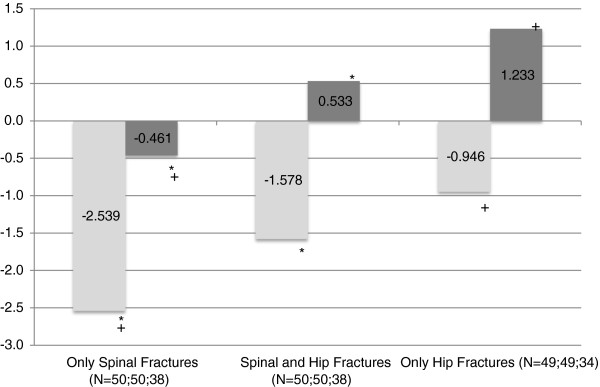
**Mean Lumbar BMD T-Scores and Z-Score.** Legend: “gray shading” BMD T-Scores, “dark gray shading” BMD Z-Scores. * indicates significant differences between the 'Only Spinal Fractures' group and 'Spinal and the ‘Hip Fractures' group (p=0.002; 0.002; 0.016). + indicates significant differences between the 'Only Spinal Fractures' group and the ‘Only Hip Fractures' group (p=0.000; 0.000; 0.001).

**Figure 2:**
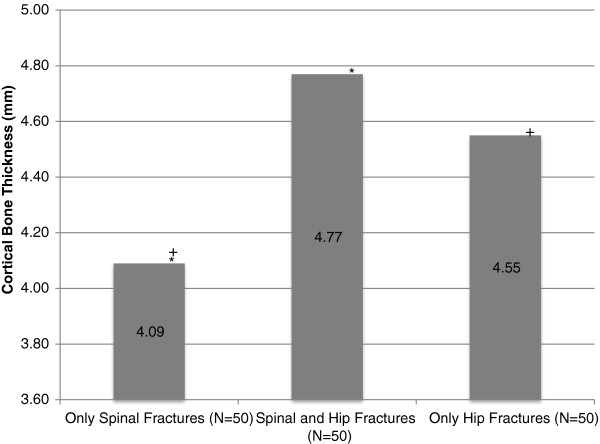
**Infero-medial Femoral Neck Cortical Bone Thickness.** * indicates significant differences between the 'Only Spinal Fractures' group and the 'Spinal and Hip Fractures' group (p=0.000). + indicates significant differences between the 'Only Spinal Fractures' group and the ‘Only Hip Fractures' group (p=0.040).

Patients with only spinal fractures had a significantly lower lumbar t-score than those presenting with hip fractures (with or without spinal fractures) (−2.54 vs. -1.27 p<0.001). Twenty-three, 16 and 23 spinal fracture only patients, hip and spinal fracture patients and hip fracture only patients respectively had degenerative changes noted on the x-ray report and 9, 8, and 7 of these respectively were reported as showing sclerosis, but the spinal BMD was not higher in these patients and their exclusion did not affect the results. Spinal fracture only patients also have a tendency to a lower hip density, reaching significance only for the intertrochanteric (50% trabecular bone) region (−2.17 vs −1.70 p< 0.05 (Table 1)). Generally, those individuals presenting with only vertebral fractures have a lower BMD t-score at all sites than those presenting with hip and spinal fractures, who, in turn, have a lower BMD t-score than those presenting with only hip fractures and no spinal fractures (Figure 1). The femoral neck t-score is the only region that does not follow this trend. Similarly, direct measurement of the femoral infero-medial cortical bone thickness shows it to be lower in the spinal fracture only patients as compared to both hip fracture groups combined (4.09 vs.4.66mm: p<0.001). The two hip fracture groups, with or without spinal fractures, have similar cortical thickness (Figure 2). Comparison of the two hip fracture groups shows that those with a spinal fracture have lower trabecular site t-scores (spine, total hip, intertrochanteric and trochanteric) but similar cortical bone measurements (neck of femur and cortical bone thickness) though none of the comparisons reached significance (Table 1).

Cortical bone thickness correlates significantly with femoral neck BMD across all subjects (r=0.36 p<0.001) and for men and women separately (r=0.41 for men and 0.45 for women p<0.023 and P<0.001 respectively).

Neither the hip axis length, femoral neck length nor femoral neck width showed any difference between groups (Table 1). Femoral neck length and hip axis length correlated strongly with femoral neck width (r= 0.71 and 0.78 respectively, both p<0.001) suggesting these are measures of bone size and perhaps subject size. The correlations for each gender separately were also highly significant with coefficients of around 0.64. Both hip length measurements and the neck width correlated with the sum of vertebral body heights in the hip fracture only female patients (there being too few men for meaningful analysis), this being the group without vertebral damage, again suggesting this is a patient size issue (for femoral neck length, r=0.48, p<0.001; for hip axis length r=0.46 p=0.002 and for femoral neck width, r=0.44 p=0.004). Interestingly the cortical bone thickness was not correlated with any of the size measurements, suggesting this is independent of size. The ratio of femoral neck length to femoral neck width was compared to see if it is those with a width which was relatively low compared to length (that is, a long and narrow neck) that are at risk but there was no difference between the groups.

When the BMD for the femoral neck is plotted on the CAROC graphs (3) of femoral neck t-score against age approximately one quarter of patients were of an age that was beyond the graphs’ limits. However, we attempted to extrapolate the graphs beyond the 85 year age limit, and found only a minority of women with hip fracture (approximately one third) and one out of every 20 men fell, on the basis of BMD alone, into the high risk category. Interestingly, the presence of spinal fractures did not increase the proportion of hip fracture patients falling into the high risk category when defined by the femoral neck t-score. For those with spinal fracture alone, only 14 (13 female and 1 male) fell into the high risk zone on the CAROC graph. On the other hand, of those with spinal fractures alone, 50% fell below a spinal t-score of −2.5, while 30% of those with hip and spinal fracture did so, and only 18% of those with a hip fracture alone. For a femoral neck t-score of −2.5, the respective numbers are 60%, 43% and 45% respectively.

### The location and severity of vertebral fractures

**Figure 3:**
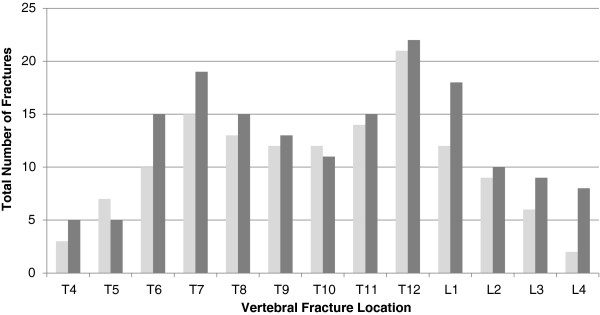
**Number of Fractures by Vertebral location.** Legend: “gray shading” Only Spinal Fractures, “dark gray shading” Spinal Fractures and Hip Fractures.

Patients presenting with spinal fractures have a higher number of spinal fractures than those with hip fracture and spinal fractures (165 vs. 138 respectively) and as a result both the spinal deformity index and the spinal fracture index are higher in the vertebral fracture alone group (5.80+/− 3.44 vs 4.25 +/− 2.50 and 0.43+/−0.26 vs. 0.327+/−0.19, respectively, p=0.029 for both) but the individual fractures are of similar severity (1.728 vs. 1.66 ns.). However, as shown in Figure 3, both groups seem to follow the same bimodal trend with the majority of fractures seen in the mid-thoracic region (T6-T8) and the thoracic/lumbar junction (T12-L1).

## Discussion

This study compared three groups of fracture patients: those with spinal fractures and no hip fractures, those with both spinal and hip fractures and those with hip fractures and no spinal fractures. Our analysis has attempted to sort out the relative contributions of trabecular and cortical bone deficiency to the different fractures and we were particularly interested to see if the presence of vertebral fractures in the ihip fracture population might identify a group with deficient trabecular bone and so be a group who could be responsive to treatment, as opposed to being a group in whom the presence of vertebral fractures was simply another reflection of their falling and hence more traumatic in nature. Patients presenting to the osteoporosis clinic with spinal fractures have generally more deficient bones than those of similar age presenting with hip fractures, including a tendency to a lower hip density. In most measurements the spinal fracture only patients have the lowest bones mass and the hip fracture patients without spinal fractures the highest and those with both spinal and hip fracture in between. This is clear for the spinal density but a similar, though less marked, trend is seen for the hip density measurements that reflect trabecular bone, and less again for the cortical site of the neck of femur. Additionally, patients presenting with spinal fractures only, tend to have both a higher number of spinal fractures than those presenting with a hip fracture and spinal fractures, although the severity of the individual fractures and the distribution of the fractures in the spine is similar. The bimodal trend of fracture location with an increased frequency around T7-T8 and another peak around T12-L1 is consistent with previous findings [16]. This pattern was not affected by the presence of hip fractures. We had hypothesised that if the vertebral fractures had been more related to trauma (falling) in the hip fracture population, there might have been a trend towards more fractures in the lower spine, the site most vulnerable to traumatic damage [13]. This appears not to be the case. These patients appear to have trabecular osteoporosis though perhaps of a milder degree than the spinal fracture only patients.

Most studies evaluating the effectiveness of osteoporosis medications have focused on patients with poor trabecular bone, as reflected in low spinal density, with or without spinal fractures, and treated them with antiresorptives which are known to mostly act on trabecular bone. In general the effect on the prevention of further vertebral fractures has been impressive while the effect on non-vertebral fractures has been less so. Few studies have focused directly on the prevention of non-vertebral fractures in less selected patients and those that have, have not been very successful. Black and colleagues showed the presence of prevalent vertebral fractures indicated a high risk for future hip fracture whereas in the study conducted by McClung, the younger 70–79 year old group showed no benefit of treatment if the patients had no prevalent vertebral fractures [17,18]. Likewise, recruiting older (>80 year old) patients on the basis of falling risk, with, in most cases, no reference to bone density, and treating with risedronate, had little effect on subsequent hip fracture rate [18]. Although some of these patients had reduced bone density this was femoral neck bone density, a measure of cortical bone, and there was no significant reduction in femoral fracture with treatment.

It also appears that recruiting patients on the basis of a prior hip fracture without regard to trabecular bone status has limited benefit in the prevention of further hip fractures. For example, the zoledronic acid study of fracture prevention in hip fracture patients showed greater ability to prevent further (clinical) spine fractures and a more modest and non-significant benefit in the prevention of further hip fractures [19]. Unfortunately the spinal state of these patients at the start of the study is unknown. In the other study with zoledronic acid, the fracture prevention study, although the bone density qualification for the study was on the basis of the femoral neck measurement, 63% had baseline vertebral fractures suggesting significant prevalence of trabecular bone deficiency, and a reduction in hip fractures was seen [20].

If it is true that the presence or absence of trabecular bone mass reduction has an influence on the likelihood of response to treatment, it does not mean that trabecular bone loss is required for a hip fracture to occur, it simply increases the risk and may influence the type of fracture that occurs. Although the definition of osteoporosis has evolved to a risk paradigm, the notion of a t-score of −2.5 representing a significantly low BMD has to some extent survived, particularly at the spine site [3]. Only a minority of our patients with a hip fracture had a spinal BMD t-score below this osteoporotic threshold, even fewer if there were no spinal fractures. Our sample is not random, and is in fact biased toward the spinal fracture subjects, as the presence of spinal fractures in the hip fracture population is likely less than the 50% we selected here. Nonetheless, using spinal BMD and a cut point of t-score of −2.5 only 30% of those with hip and spinal fractures and 18% of those with hip fracture without spinal fractures fell below that point, while 50% of those with spinal fractures alone did so. In addition we find that most women and all men in our study would not have been in the high risk category using the CAROC paradigm on the basis of femoral neck BMD alone although the CAROC graph for men is unusual in showing no age-related rise in fracture risk. We were not able to calculate prior risk on the FRAX model as we had not gathered the extra information required by that model. These observations are similar to those reported by others, and the observations of Stone and colleagues who found low bone density to explain only a modest number of fractures [21-23]. These observations speak against the automatic labelling of hip fracture patients as osteoporotic and presumably reflect the large number of outcomes (hip fractures) that originate in the larger population of people at relatively low or moderate risk, have fairly good bones, but fall in such a way as to endanger the integrity of the hip bone. Although the absolute risk in such patients is low, their greater numbers likely ensure they contribute substantially to the overall hip fracture numbers but treatment of such low risk subjects may not be of value as far as the hip fracture prevention is concerned. Certainly treating those with relatively good femoral neck bone density was of no benefit [24]. If preventing a hip fracture in such patients with bisphosphonates is relatively unsuccessful then preventing a second hip fracture in such patients is also likely to be unsuccessful.

A prior “fragility fracture” is certainly associated with an increased risk of a further one, but this may be on the basis of a risk of recurrent falling. If there is no second fall, there will be no second fracture. Of note is the finding that the increased risk of a second fracture in those who have experienced a first fragility fracture, is largely independent of bone density, and even more so in older age, pointing to falling as the predominant risk factor [25]. Likewise in their study of the time for people with a certain bone density at the hip to cross the osteoporosis threshold, Gourley found that the presence of a prior fracture had no effect [26].

It is notable that in our study the z-scores in the hip fracture patients without spinal fracture are normal (that is, above or close to zero) indicating no greater deficiency in skeletal mass in these patients than in the age and gender matched normative databases of the particular densitometers (Table 1). Given the dynamics of falling in old age, with a greater tendency to fall from a static position backwards on to the greater trochanter, it may be that all older people are at risk of a hip fracture should they fall in this manner. However those with trabecular bone deficiency will be at higher risk as the trabecular bone contributes 50% of the hip mass. The relative degree of loss of trabecular versus cortical bone loss may be important in determining the nature of the hip fracture. Thus the intertrochanteric site has more trabecular bone than the subcapital site and patients with IT fractures have lower spinal density [27]. It has been suggested that lower trabecular bone mass places the intertrochanteric site at risk, and, as it collapses more readily, this protects the subcapital site from the transmitted force [28]. Other studies have found that femoral neck length was greater in those who suffered a subcapital fracture [29-32]. Our data suggests this may be related simply to greater person height as neck length, neck width and summed vertebral body heights are related. Taller people may be at more risk of a hip fracture in a fall with trauma, rather than bone deficiency, being the predominant cause and leading to a greater tendency to subcapital fractures [33]. Our study suggests that these architectural considerations are independent of the presence or otherwise of trabecular bone status.

A potential weakness of the study is that the patients with hip fracture are a selected group of convenience. Patients missed, as noted above, are those who are younger and go directly home and those who go directly to LTC, who tend to be older. However as our comparison is with elderly ambulatory osteoporotic patients attending the osteoporosis clinic, the exclusion of a younger group, and an older, particularly frail group, is not inappropriate. Our study includes both genders on the assumption that hip fractures in either sex will show similar deficiencies regardless of gender. Although there are more women in the group with hip fracture and no spinal fracture, the analysis shows this to be group with the best bones, a finding which would be biased against by a predominance of women. On the other hand the spinal fracture alone group were slightly younger, but they turned out to have the most deficient bones. The analysis was re-run on the women alone. The smaller numbers made some comparisons fall below the significance level but overall the trends were unchanged.

## Conclusion

It appears that the risk of a hip fracture is a complex interaction of falling, person height, hip architecture and cortical and trabecular bone status, varying combinations of which determine the risk overall and, perhaps, the particular type of hip fracture sustained. It may be that only one of these factors, trabecular bone status, predicts a response to the current antiresorptives which are predominantly active on trabecular bone and the presence of vertebral fractures may be a simple indicator of such a state. More detailed reporting of the randomized controlled studies, in particular regarding the trabecular and spinal fracture status and the nature of the hip fractures that occur and the different responses to treatment in these various sub-groups, might bring better understanding of this phenomenon.

## Competing interests

The authors declare that they have no competing interests.

## Authors’ contributions

RC conceived of the study and directed the data collection and wrote the manuscript. LC completed the data collection, performed the analysis and reviewed the manuscript. Both authors read and approved the final manuscript.

## Pre-publication history

The pre-publication history for this paper can be accessed here:

http://www.biomedcentral.com/1471-2474/14/68/prepub
